# Partially absorbable mesh or native tissue repair for pelvic organ prolapse: a randomized controlled trial

**DOI:** 10.1007/s00192-018-3757-5

**Published:** 2018-08-29

**Authors:** Pieternel Steures, Alfredo L. Milani, Deliana A. van Rumpt-van de Geest, Kirsten B. Kluivers, Mariëlla I. J. Withagen

**Affiliations:** 1 Department of Obstetrics and Gynaecology, Reinier de Graaf Gasthuis, Delft, The Netherlands; 2 Department of Obstetrics and Gynaecology, Jeroen Bosch Ziekenhuis, Postbus 90153, 5200 ME Den Bosch, The Netherlands; 3 Department of Obstetrics and Gynaecology, Radboud University Medical Center, Nijmegen, The Netherlands; 4 Department of Obstetrics and Gynaecology, University Medical Center Utrecht, Utrecht, The Netherlands

**Keywords:** Adverse events, Anatomical success, Composite outcome, Native tissue repair, Partially absorbable mesh, Primary, Pelvic organ prolapse

## Abstract

**Introduction and hypothesis:**

The objective was to compare medium-term efficacy and safety of a partially absorbable mesh kit and native tissue repair in pelvic organ prolapse (POP).

**Materials and methods:**

Women with primary POP stage ≥ II were randomized to transvaginal trocar-guided partially absorbable mesh (81 women) or native tissue repair (82 women). Primary outcome was overall anatomical success (POP < stage II) at 24 months. Secondary outcomes were composite success, global improvement, and adverse events.

**Results:**

Sixty-nine (85%) of the women allocated to partially absorbable mesh underwent mesh surgery; 8 (10%) crossed over to native tissue repair and 4 women (5%) withdrew from the study. Eighty (98%) of the women allocated to native tissue repair underwent the assigned treatment and 2 (2%) withdrew. Twenty-four months later, 140 surgically treated women (89%) demonstrated an overall anatomical success of 39%; 45% (32 out of 71 women) for mesh, and 32% (22 out of 69) for native tissue repair (RR 1.4, 95% CI 0.92 to 2.2). Composite success was 88 and 73% respectively (RR: 1.1, 95% CI 0.93 to 1.4). There was global improvement in 86% (48 out of 56 women) in the mesh group and in 77% (47 out of 60 women) in the native tissue group (RR: 1.1, 95% CI 0.92 to 1.3). Four women were diagnosed with mesh exposure at 2 years (6%).

**Conclusion:**

At 24 months, no significant anatomical or composite benefit of partially absorbable mesh over native tissue repair could be demonstrated in women who had been surgically treated for primary POP.

## Introduction

Pelvic organ prolapse (POP) occurs in up to 50% of parous women [1]. The lifetime risk of undergoing surgery for POP in the general population is estimated to be between 13 and 19% [2, 3]. Anatomical recurrence rates after primary repair of POP using patients’ own native tissues vary from 31 to 59% [4, 5]. These high rates have led to a continuous search for more durable solutions in the surgical repair of POP. Since 2002, clinical research began into the use of synthetic mesh in POP, inspired by promising results in inguinal hernia surgery and surgery for stress-urinary incontinence [6–15]. At the same time, the use of these prosthetic meshes in vaginal POP surgery in daily clinical practice increased enormously, whereas no results from scientific research were yet available [16]. The use of prosthetic mesh also caused mesh-related adverse events [17]. Among the most prevalent adverse events are vaginal mesh exposure and pelvic pain and/or dyspareunia. Long-term follow-up of a randomized controlled trial with a synthetic mesh kit demonstrated a cumulative mesh exposure rate of 42% at 7 years [18]. To reduce both POP recurrence rates and the frequency of adverse events that accompanied the use of non-absorbable synthetic mesh, the rationale to adopt a partially absorbable, lighter-weight mesh with improved directional elastic properties arose, with the intent of increased biocompatibility. A prospective cohort study of 127 women treated with a trocar-guided, partially absorbable mesh kit demonstrated favorable outcomes in efficacy and safety at 1 year [19]. However, any comparison with conventional native tissue repair was lacking. Therefore, this randomized controlled trial was designed to assess and compare the efficacy and safety of that trocar-guided, partially absorbable mesh kit with those of conventional native tissue repair in women with primary POP.

## Materials and methods

The study was conducted in five teaching hospitals in the Netherlands, between January 2011 and February 2013; the study was approved on 29 July 2010, by the Medical Ethics Committee, region Arnhem-Nijmegen, the Netherlands, and by all local ethics committees of the participating hospitals. The trial was registered at clinical.gov, number NCT 02231099. Consecutive women with symptomatic POP (≥ stage II) were asked to participate and underwent a systematic work-up, which included medical history, completion of urogynecological questionnaires (measuring generic and disease-related quality-of-life and sexual functioning), that comprised the Dutch validated Urogenital Distress Inventory (UDI), Defecatory Distress Inventory (DDI), Incontinence Impact Questionnaire (IIQ), Pelvic Organ Prolapse/Urinary Incontinence Sexual Function questionnaire (PISQ12), and routine gynecological investigation including pelvic organ prolapse quantification (POP-Q) [20–23]. Exclusion criteria were previous vaginal POP repair or mid-urethral sling surgery, a compromised immune system or malignancy. After written informed consent had been obtained, women were randomly assigned to either trocar-guided, partially absorbable mesh insertion (Gynecare Prolift+M Pelvic Floor Repair System, referred to as Prolift+M; Ethicon, Somerville, NJ, USA) or conventional native tissue repair. Women were informed about the treatment they had been assigned to and surgery was scheduled. The randomization sequence was computer generated in balanced block multiples, stratified by center. The allocation was centralized: inclusions could not be changed or removed. The participating gynecologists were all surgeons experienced in pelvic floor reconstruction and vaginal mesh insertion.

Mesh insertions were performed as described by Fatton et al. [24]. The insertion of Prolift +M™ was similar to the insertion of the non-absorbable variant Prolift™. The composition of the mesh of Prolift +M™ (M stands for Monocryl) is entirely different though. Prolift + M™ is composed of a 50-50 blend of monofilament non-absorbable polypropylene mesh and absorbable poliglecaprone 25. Before absorption, this mesh weighs 57 g/m^2^. Full absorption after 90–120 days results in a final weight of 31 g/m^2^, as opposed to the 45 g/m^2^ of the original non-absorbable polypropylene mesh. Because of warp knitting, this mesh provides increased elasticity in the longitudinal direction and has larger pores than the non-absorbable variant, to allow more tissue ingrowth [18]. After mesh insertion, no resection of redundant vaginal tissues was performed. Simultaneous hysterectomies or T-incisions were not allowed to minimize the risk of mesh exposure [24]. Additional native tissue surgery for restoration of level I support (sacrospinous fixation of the uterus, for example) was permitted.

Native tissue repairs were performed as follows: anterior colporrhaphy; midline anterior vaginal incision, dissection of the vaginal epithelial layer from the fibromuscular layer, midline plication of the fibromuscular layer with Vicryl 2–0, optional excision of redundant vaginal mucosa, and closure of the vagina with a running absorbable Vicryl 2–0 suture. For the apical compartment (uterus, vaginal vault or cervix) a vaginal hysterectomy with vault suspension, modified Manchester Fothergill procedure, uterosacral vaginal suspension (McCall procedure), or sacrospinous ligament fixation was allowed. Posterior colporrhaphy was performed through a posterior vaginal midline incision, dissection of the vaginal epithelial layer from the fibromuscular layer, midline plication of the fibromuscular layer with Vicryl 2–0, optional excision of excess vaginal mucosa, and incision closure with Vicryl 2–0. Perineoplasty was optional but not recommended. Perioperative antibiotic prophylaxis and postoperative thrombosis prophylaxis were performed in both groups according to local protocols. An indwelling urinary catheter and vaginal gauze pack were left for one night.

Postoperative evaluations were performed during the hospital stay, at 6 weeks, and at 6, 12, and 24 months after surgery. In most women (72%), 2-year follow-up assessments were performed by the operating gynecologist; in the remaining group (28%), this was performed by another gynecologist who was not blinded to the treatment. Women underwent a gynecological examination at 6 weeks, which was combined with POP-Q at 6, 12, and 24 months’ follow-up. Women completed the same validated urogynecological questionnaires as at baseline with the addition of the Patient Global Impression of Improvement (PGI-I) questionnaire [25]. The PGI-I is a single question with a seven-point Likert scale, asking the woman to compare her condition at the moment she answered this question with how she felt before surgery on a scale from 1, very much better, to 7, very much worse. Also, in women with re-surgery for POP or complications, the PGI-I compares her condition with how she was before the initial surgery.

Primary outcome was overall anatomical success, defined as POP < stage II in all three vaginal compartments at 24 months’ follow-up. Secondary outcomes were composite success, defined as POP ≤ hymen, absence of bulge symptoms, and no re-intervention for POP, duration of surgery, estimated blood loss, length of hospital stay, global improvement as measured by the PGI-I, and adverse events [23]. Improvement was considered present if a participant responded at least “better” to the question: “how is your post-operative condition compared with your condition before surgery?” Stress urinary incontinence (SUI) was considered present in case a woman responded, “yes, moderately to quite a bit” to the question “do you experience urinary leakage during physical activity, coughing, or sneezing?” Dyspareunia was considered present if a woman responded, “yes, moderately to quite a bit” to the question “do you experience pain during intercourse?” Pelvic pain was considered present if a woman responded, “yes, moderately to quite a bit” to the question “do you experience pain in the lower abdomen or genital region?”

Sample size calculation was based on the assumption of the superiority of surgery with partial absorbable mesh: a POP recurrence rate of 30% in the native tissue group (success rate: 70%) and less than 12% in the Prolift+M™ group (success rate ≥ 88%) [6–12]. To demonstrate a significant difference, 76 women would be required in each group (α=5%, β=80%). Anticipating a dropout rate of 15%, a total of 176 women would be required.

Data were analyzed according to the intention-to-treat principle. Treatment effects of surgery were calculated as relative risks (RRs) with 95% confidence intervals (95% CIs). A “per protocol” analysis was performed to compare separate results of mesh and native tissue. A benefit:risk ratio was added and calculated as follows: anatomical success percentage divided by the cumulative risk of major adverse events, such as blood loss more than 500 ml, mesh exposure, de novo pain, and de novo dyspareunia. Continuous variables were compared using the independent samples *t* test or Mann–Whitney *U* test where appropriate. Categorical variables were compared using paired-samples *t* test or the Wilcoxon signed rank test where appropriate.

## Results

**Fig. 1:**
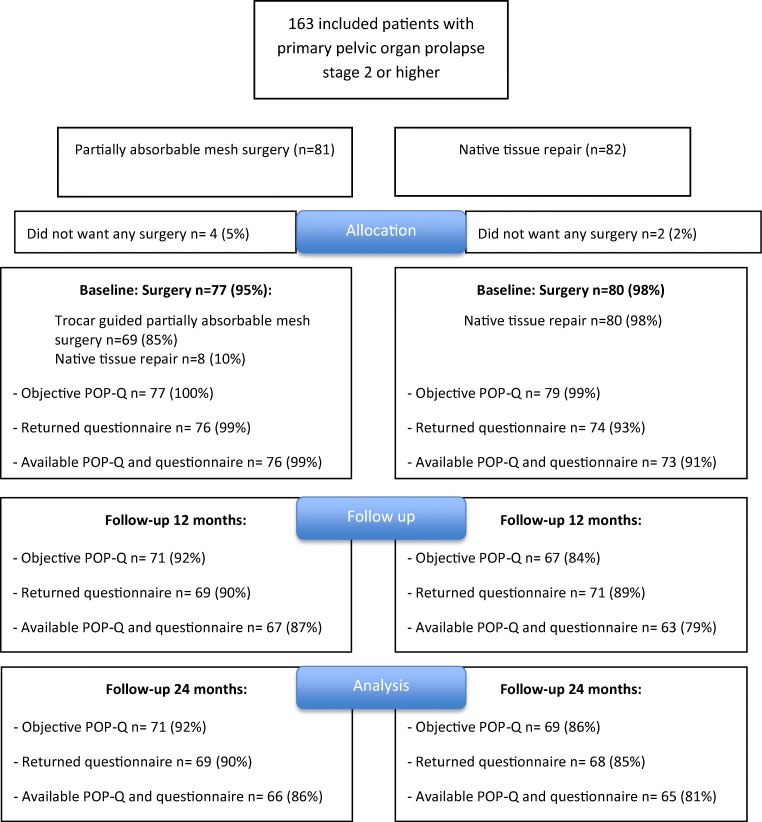
Consolidated Standards of Reporting Trials (CONSORT) flowchart of randomization and follow-up. *POP-Q* pelvic organ prolapse quantification system

A total of 163 women were randomly assigned: 81 women to the partially absorbable mesh group and 82 women to native tissue repair group (see the flowchart in Fig. 1).

In December 2012, the broadcast of a consumer television program at prime time on the complications of vaginal mesh surgery made the inclusion of participants increasingly difficult; some women no longer wanted surgery, with or without vaginal mesh. Six women, already randomized and waiting for surgery, withdrew from any surgery (mesh or native tissue repair) and from further participation in the study. Those women have been excluded from final analyses and have therefore not been included in the baseline characteristics. Women were recruited for this trial at Radboud University Medical Center, Nijmegen (13%), Reinier de Graaf Gasthuis, Delft (50%), Gelre hospital, Apeldoorn (11%), Zaans Medical Center, Zaandam (5%) and Isala Klinieken, Zwolle (21%).

A total of 8 women allocated to the mesh arm (10%) refused mesh treatment and crossed over at their request to native tissue repair. Those women remained in the final analyses. According to the intention-to-treat principle, these 8 women were analyzed in the mesh group. In February 2013, when further recruitment appeared no longer possible, it was decided to close the trial prematurely. The pre-specified sample size could therefore not be reached.

At 24 months, the follow-up rate was 89% (140 women of the 163 randomized women); 71 women (92%) in the mesh group and 69 (86%) women in the native tissue repair group. Of the 71 women in the mesh group with complete 24-month follow-up, 64 women (83%) had an operation with mesh.

Baseline characteristics of the two groups were comparable and are shown in Table 1. Included women had a POP-Q stage 4, 3 or 2 prolapse. In the women with POP-Q stage 2, all had pelvic organ descent reaching the hymen or further. The type of surgery is specified in Table 2. Overall anatomical success at 24 months was 45% (32 out of 71) for mesh and 32% (22 out of 69) for native tissue (RR 1.4, 95% CI 0.92 to 2.2; Tables 3 and 4). At 1 year, anatomical success was significantly in favor of the mesh group (RR 1.7, 95% CI 1.1 to 2.8). At 2-year follow-up, this difference had disappeared.

**Table 1:** Baseline characteristics

	Partially absorbable mesh (*n* = 77)	Native tissue repair (*n* = 80)	*p* value
Age in years^a^	65.0 ± 10.5	65.4 ± 8.5	0.81
Parity^b^	3.0 (1 to 6)	3.0 (0 to 8)	0.56
BMI^a^	26.7 ± 3.6	26 ± 3.2	0.22
Comorbidity^c^	43 (56)	41 (51)	0.63
Previous hysterectomy not for POP			
Abdominal hysterectomy^c^	8 (10)	7 (8.8)	0.76
Vaginal hysterectomy^c^	2 (2.6)	3 (3.8)	0.71
Overall POPQ-stage^c^			
II	21 (27)	18 (23)	0.58
III	56 (73)	61 (76)	0.71
IV	0 (0)	1 (1)	1.0
Prolapse anterior compartment (Ba)^a^	2 (−3 to 6)	2 (−3 to 8)	0.39
Prolapse posterior compartment (Bp)^b^	−2 (−3 to 5)	−2 (−3 to 8)	0.92
Prolapse apical compartment (C)^b^	−3 (−8 to 6)	−2 (−9 to 8)	0.28
Sensation of bulge	68/75 (91)	66/72 (92)	0.83
Treatment anterior compartment	66 (86)	68 (85)	1.0
Treatment posterior compartment	30 (39)	29 (36)	0.74
Treatment apical compartment	48 (62)	74 (91)	0.02

Data presented as means (± standard deviation)^a^, median (range)^b^ or number of patients (%)^c^

*BMI* body mass index, *POP-Q* pelvic organ prolapse quantification system, *CI* confidence interval, *POP* pelvic organ prolapse

**Table 2:** Peri- and postoperative data

	Partially absorbable mesh (*n* = 77)	Native tissue repair (*n* = 80)	*p* value
Anterior Prolift™^a^	48 (62)	0	
Posterior Prolift™^a^	12 (16)	0	
Anterior + posterior Prolift™^a^	6 (8)	0	
Total Prolift™^a^	3 (4)	0	
Cross-over	8 (10) 4× sacrospinous fixation with anterior colporrhaphy 1× sacrospinous fixation with anterior and posterior colporrhaphy 1× vaginal hysterectomy with anterior colporrhaphy 1× vaginal hysterectomy with anterior and posterior colporrhaphy 1× posterior colporrhaphy	0	
(Concomitant) surgery^a^	45 (58)		
Vaginal hysterectomy	0	9 (11)	
Anterior colporrhaphy	2 (posterior group)	68 (85)	
Posterior colporrhaphy	6 (anterior group)	29 (36)	
Perineoplasty	2 (anterior group)	1 (1)	
Manchester Fothergill	1 (anterior group)	2 (2.5)	
TVT-O	0	2 (2.5)	
Sacrospinous ligament fixation	36 (anterior group)	53 (66)	
Portio amputation	1 (anterior group)	2 (2.5)	
Uterosacral vaginal suspension		8 (10)	
Spinal analgesia^a^	46/74 (62)	52/78 (67)	0.61
General analgesia	28/74 (38)	26/78 (33)	0.61
Operating time (min)^b^	55 (26–140)	40 (20–150)	0.10
Blood loss (ml)^b^	50 (0–800)	50 (0–350)	0.043
Duration urinary catheter (days)^b^	1 (1–7)	1 (1–9)	0.687
Hospital stay (days)^b^	2 (1–13)	2 (1–4)	0.609

Data presented as number of patients (%)^a^ or median (range)^b^

**Table 3:** Primary and secondary outcomes at 12 and 24 months

Intention to treat	Partially absorbable mesh (*n*=77)	Native tissue repair (*n*=80)	RR (95%CI)
12 months			
Primary outcome			
Overall < stage II (< -1 cm)	33/71 (46)	18/67 (27)	1.73 (1.1 to 2.8)
Secondary outcomes			
Re-operation for POP	1/71	1/69	^a^
Sensation of bulge	4/66 (6)	6/69 (9)	0.70 (0.21 to 2.36)
POP ≤ hymen	68/71 (96)	58/67 (87)	1.1 (0.995 to 1.23)
Composite success	50/58 (86)	48/62 (86)	1.1 (0.94 to 1.32)
PGI-I (much to very much better)	49/55 (89)	49/59 (83)	1.07 (0.93 to 1.24)
24 months			
Primary outcome			
Overall < stage II (< -1 cm)	32/71 (45)	22/69 (32)	1.4 (0.92 to 2.2)
Secondary outcomes			
Re-operation for POP	5/75 (7)	1/77 (1)	5.1 (0.6 to 43)
Sensation of bulge	5/70 (7)	10/64 (16)	0.46 (0.17 to 1.3)
POP ≤ hymen	70/71 (99)	62/69 (90)	1.1 (1.0 to 1.2)
Composite success	49/56 (88)	43/59 (73)	1.1 (0.93 to 1.4)
PGI-I (much to very much better)	54/63 (86)	41/53 (77)	1.11 (0.93 to 1.32)

Data presented as numbers (%)

*RR* relative risk, *95%CI* 95% confidence interval, *PGI-I* Patient Global Impression of Improvement, *POP* pelvic organ prolapse, *composite success* POP ≤ hymen, no sensation of bulge, no re-operation for POP

^a^Too few cases to allow estimation of the OR or RR

**Table 4:** Per protocol analysis

Per protocol analysis	Partially absorbable mesh (*n*=69)	Native tissue repair (*n*=88)	RR (95% CI)
24 months			
Primary outcome: success rate			
Overall < stage II (< -1 cm)	30/64 (47)	24/76 (32)	1.48 (0.97 to 2.3)
Secondary outcomes			
Re-operation for POP	5/68 (7.4)	1/84 (1.2)	6.2 (0.74 to 52)
Sensation of bulge	4/63 (6.3)	11/71 (15)	0.41 (0.14 to 1.2)
POP ≤ hymen	64/64 (100)	68/76 (89)	1.12 (1.04 to 1.2)
Composite success	46/55 (84)	46/63 (73)	1.15 (0.95 to 1.4)
PGI-I (much to very much better)	48/56 (86)	47/60 (78)	1.09 (0.92 to 1.3)

Data presented as numbers (%)

*RR* relative risk, *95%CI* 95% confidence interval, *PGI-I* Patient Global Impression of Improvement, *POP* pelvic organ prolapse, *composite success* POP ≤ hymen, no sensation of bulge, no re-operation for POP

Secondary outcomes are shown in Table 3: re-surgery for POP was performed in 5 women (7%) of the mesh group within 2 years. Four of these 5 women (80%) had a symptomatic POP in the nontreated vaginal compartment. The fifth woman underwent two additional POP surgeries; index surgery was an anterior and posterior mesh, recurrent treatment was laparoscopic hysterosacropexy, and was followed 6 months later by an amputation of the cervix. One woman (1%) in the native tissue repair group underwent a laparoscopic hysterosacropexy for recurrence in the treated anterior and apical compartments (7 versus 1%, RR: 5.1, 95% CI 0.6 to 43). The PGI-I scores of these 6 women who needed re-surgery showed; no change in 1 woman (much better), improved in 1 woman from no difference to a little better, and improved in 2 women to much better. The PGI-I deteriorated in 2 women from very much better to much better and much better to a little better.

Composite success rates were not significantly different: 88% for the mesh group versus 73% for the native tissue repair group (RR 1.1, 95% CI 0.93 to 1.4). Global impression of improvement was 86% for women with mesh and 77% for women after native tissue repair and was not significantly different either (RR: 1.1, 95% CI 0.93 to 1.3).

Per protocol analysis did not alter these outcomes (Table 4). In the intention-to-treat analyses and in the per protocol analyses, the only variable that appeared significantly in favor of mesh was the outcome variable “no POP beyond the hymen in any compartment” (Tables 3, 4).

The benefit/risk ratio for partially absorbable mesh was 3.6; anatomical success (45%) divided by the cumulative percentage of “major” adverse events (blood loss >500 ml [1 out of 77 = 1.3%], mesh exposure [4 out of 71 = 5.6%], de novo pain [1 out of 75 = 1.3%], and de novo dyspareunia [3 out of 72 = 4.2%]). For native tissue repair this ratio was 2.7; anatomical success (32%) divided by the risk for de novo pain [5 out of 73 = 7%] and the risk for de novo dyspareunia [3 out of 60 = 5%]). The difference was nonsignificant (RR 1.1, 95% CI 0.9 to 1.4).

Peri- and postoperative characteristics were not significantly different (Table 2). Median blood loss was 50 ml in both groups; however, because of the severe hemorrhage in one woman in the mesh group (800 ml), there was a significant difference between the groups (*p* value of 0.04; Table 2). Her recovery, though, was uneventful and she left the hospital the day after surgery in a fair condition, without any extra medication, no re-surgery or blood transfusion. Another woman, treated with anterior mesh and sacrospinous ligament fixation, suffered a paravaginal hematoma that was noticed during surgery. She was prophylactically admitted to the intensive care unit for observation: no embolization was necessary; she received an indwelling urinary catheter for 7 days, owing to temporary urinary retention. After 13 days she was able to leave the hospital in a good condition. The hematoma had spontaneously resolved by 6 weeks. In this patient, no re-surgery was needed; no de novo pain was present at 24 months. Temporary urinary retention was the most common adverse event in both groups, although this occurred significantly more often in the mesh group; 16 (21%) versus 7 (9%) in the native tissue group respectively (RR: 2.7, 95% CI 1.2 to 6.3). Normal micturition was restored spontaneously in all these women.

In 4 out of 71 women treated with mesh (6%), a mesh exposure was detected, in 3 of them at the 6-month follow-up and in 1 other woman at 24 months. All exposures were seen in the anterior compartment. Two of the 4 women were asymptomatic; the other 2 had symptoms of vaginal discharge. Two women were treated successfully with local estrogens. One woman needed supplementary surgery, which was performed in the operating theater, and the exposure was successfully excised. One asymptomatic woman did not want any treatment. Three of these 4 women completed the PGI-I and mentioned much or very much improvement compared with the situation before the initial POP surgery.

Forty-two out of 75 women (56%) in the mesh and 35 out of 73 women (48%) in the native tissue group had reported pain in the lower abdomen or in the genital area at baseline. Thirteen out of 67 women (16%) in the mesh group and 19 out of 66 women (29%) in the native tissue group reported pain in the lower abdomen or genital region at 24 months (RR: 0.67, 95% CI 0.36 to 1.3; Table 5). De novo pain was rare in both groups: at 2 years 1 woman in the mesh group (1.3%) and 5 women in the native tissue repair group (7%) reported de novo pain (RR: 0.19, 95% CI 0.02 to 1.6).

**Table 5:** Adverse events

	Partially absorbable mesh (*n*=77)	Native tissue repair (*n*=80)	RR (95%CI)
Bladder perforation	0	0	–
Hematoma	1	0	–
Temporary urinary retention	16 (21)	7 (9)	2.7 (1.2 to 6.3)
Cumulative mesh exposure	4/71 (6)		
6 months	3/66		
12 months	0/71		
24 months	1/71		
Pain (lower abdomen or vulva/vaginal)			
At baseline	42/75 (56)	35/73 (48)	1.2 (0.85 to 1.6)
Remaining pain after surgery at 12 months	11/38 (29)	15/29 (52)	0.56 (0.30 to 1.0)
De novo pain remaining at 24 months	1/75 (1.3)	5/73 (7)	0.19 (0.02 to 1.6)
Total women with pain at 24 months	13/67 (16)	19/66 (29)	0.67 (0.36 to 1.3)
Dyspareunia in the sexually active women			
At baseline	7/59 (12)	3/55 (5.5)	2.2 (0.59 to 8.0)
Remaining dyspareunia after surgery	0/5	0/3	^a^
De novo dyspareunia at 24 months	3/52 (5.8)	3/60 (5)	1.2 (0.24 to 5.5)
Stress urinary incontinence			
De novo stress incontinence at 24 months	11/72 (15)	8/68 (12)	1.3 (0.6 to 3.0)
Additional surgery (TVT-O) for de novo SI	3/11 (27)	0/8 (0)	^a^

Data presented as numbers (%)

*RR* relative risk, *95% CI* 95% confidence interval, *TVT-O* tension-free vaginal tape through the obturator foramen

^a^Too few cases to allow estimation of the OR or RR

At baseline, 59 women (77%) in the mesh group and 55 women (69%) in the native repair group had reported that they were sexually active and completed the PISQ-12 questionnaire. Seven women in the mesh group (12%) versus 3 women (6%) in the native tissue repair group reported dyspareunia at baseline. Twenty-four months after surgery, in 8 of these women, dyspareunia had resolved. At 24 months, 1 woman in the mesh group was no longer sexually active for unknown reasons and 1 woman was lost to follow-up. At 24 months, dyspareunia and de novo dyspareunia rates did not significantly differ between groups (RR: 1.2, 95% CI 0.24 to 5.5; Table 5).

Rates of de novo SUI at 24 months were not significantly different between groups either (Table 5). Eleven out of 72 women (15%) in the mesh group, and 8 out of 68 in the native repair group (12%) reported de novo SUI (RR: 1.3, 95% CI 0.6 to 3.0). In the mesh group, 3 women (4%) underwent mid-urethral sling surgery for the treatment of de novo SUI post-mesh implantation; they were all continent at 24 months.

## Discussion

This randomized controlled trial did not demonstrate an anatomical benefit of a partially absorbable mesh kit over native tissue repair at medium-term follow-up in women who were surgically treated for primary POP. There were no significant differences in composite success or in de novo pain and/or dyspareunia rates between the groups. Mesh exposure rate at 24 months was 6% and appears low compared with that for non-absorbable mesh [1].

Although at 1-year follow-up, the anatomical effect was significantly in favor of mesh, this effect had vanished at 24 months. The increased unidirectional elasticity, and the reduced amount of remaining mesh after full absorption of the poliglecaprone could have played a role in the decreasing support. Most recurrences in the mesh group, however, were detected in nontreated vaginal compartments. This phenomenon was also observed for non-absorbable mesh kits [13, 18, 26]. In our opinion, these observations support the clinical value of longer periods of follow-up in POP trials than just 12 months.

Mesh exposure was seen in 4 out of 71 women (6%). In a randomized controlled trial with non-absorbable mesh after 1 year follow-up, an exposure rate of 17% was reported and published 7-year exposure rates were 42% [18, 26]. The difference in the exposure rate may be the fact that the mesh used in this study was partially absorbable, leaving a smaller amount of non-absorbable mesh behind, and it had larger pore sizes (4.0 mm), lower density (28 g/ m^2^), and a reduced surface area, which may have led to a reduction in fibrotic reaction around the individual mesh fibers, which could have resulted in a more physiological integration of the mesh [19, 27]. This lower exposure rate may also be a result of the increased experience of the participating surgeons in this study, as all surgeons were fully trained [28].

Although the occurrence and stage of descent in the apical compartment did not differ between the groups, treatment in this compartment did differ: 48 women (62%) in the mesh group versus 74 women (91%) in the native tissue repair group (Table 1). Two women in the mesh group underwent a vaginal hysterectomy (cross-overs) and 9 women (11%) in the native tissue repair group (Table 2). The clinical approach to the treatment of the apical compartment in mesh surgery and native tissue repair seems to be different. This could have affected outcome. It has been demonstrated by Lowder et al., that adequate restoration of level I support has a profound impact on the reduction of anterior and posterior compartment prolapse [29].

The most important limitation of this study was that we were unable to reach the pre-specified sample size from the power calculation. This was because of patients’ concerns regarding mesh surgery after a national television broadcast of a consumer program at prime time, in which women who were treated with vaginal mesh reported severe pain. Their stories had a great negative impact on patients’ trust in mesh treatment and POP surgery overall. As a consequence, 8 women who were initially allocated to mesh treatment refused surgery with mesh and crossed over to the native tissue repair group. Thus, we lacked 5 women in the mesh group to reach the pre-specified power of the study. We wondered how this one-sided underpowering would have influenced the primary outcomes, and we therefore tested different sensitivity scenarios. In the case of a 32% success rate among women lost to follow-up in the native tissue group versus 100% success in the women who had been lost to follow-up in the mesh group, a borderline statistically significant benefit over native tissue repair would have been reached (RR: 1.5, 95% CI 1.0 to 2.2). In all other scenarios, however, this statistical significance would disappear.

The strength of the study is that this is the only randomized controlled trial on a partially absorbable mesh (kit) in POP surgery. The last update of the Cochrane review included 37 studies on the effectiveness and safety of mesh, with a total of 4,023 women [1]. There was not one study on partially absorbable mesh, but 25 on non-absorbable mesh, 3 on totally absorbable mesh, and 10 on biological grafts [1]. In our opinion, a partially absorbable mesh could potentially be a fair choice for further research and development, as the failure rates in the mesh group were mainly caused by de novo POP in an untreated compartment and this partially absorbable mesh was only associated with a very modest mesh exposure rate, and no higher rates of other serious adverse events compared with native tissue surgery.

Important additional research would also involve a better selection of women who could potentially benefit from (partially absorbable) mesh, e.g., selecting those with an increased risk for recurrence for POP. A prediction model for POP recurrence may facilitate this selection [30].

In conclusion, at 24 months’ follow-up, no anatomical or subjective benefit of partially absorbable mesh over native tissue repair could be demonstrated in women who were surgically treated for primary POP.
